# Impact of Co-Spray Drying with Leucine or Trileucine on Aerosol Performance, In Vitro Dissolution, and Cellular Uptake of Colistin Powder Formulations for Inhalation

**DOI:** 10.3390/pharmaceutics17020199

**Published:** 2025-02-05

**Authors:** Yijing Huang, Kinnari Santosh Arte, Chanakya D. Patil, Qi Zhou, Li Qu

**Affiliations:** Department of Industrial and Molecular Pharmaceutics, College of Pharmacy, Purdue University, West Lafayette, IN 47907, USA

**Keywords:** spray drying, dry powder inhaler, aerosol performance, dissolution, cellular uptake

## Abstract

**Background/Objective**: Surface enrichment of hydrophobic excipients via spray drying has been demonstrated as an efficient way to protect the dry powder inhaler formulations against moisture-induced deterioration in aerosol performance. However, the impact of such surface enrichment on dissolution and cellular uptake is less investigated, which can affect the safety and efficacy of dry powder inhalers (DPIs). **Methods**: In the present work, hygroscopic colistin was coated with leucine or trileucine, at different weight ratios during spray drying. All the powders were exposed to 75% relative humidity for one week. The aerosol performance was compared before and after the moisture exposure. Various solid-state characterizations, including particle size, particle morphology, crystallinity, water sorption/desorption, and surface composition, were conducted to evaluate the properties of spray-dried colistin with/without leucine or trileucine. **Results**: The results indicated that leucine or trileucine could protect the aerosol performance of spray-dried colistin against moisture deterioration. Leucine crystallized after spray drying with colistin, and such crystal leucine could further hinder water uptake when leucine was at a 20% or higher weight ratio. Trileucine did not crystallize after spray drying with colistin nor reduce the water uptake. Interestingly, trileucine showed a superior moisture protective effect to that of leucine, which could be attributed to its better surface enrichment efficiency than that of leucine due to its lower water solubility. **Conclusions**: Importantly, our results showed that the surface enrichment with leucine and trileucine did not significantly affect in vitro dissolution of colistin in the Franz cell test and cellular uptake of colistin in the H441 lung epithelium cell model, which could be attributed to small particle size and incomplete surface coverage by leucine or trileucine.

## 1. Introduction

Pulmonary delivery holds promise to efficiently target lung diseases such as asthma, chronic obstructive pulmonary disease (COPD), and bacterial lung infections [1,2,3,4]. Dry powder inhalers (DPIs) utilize a device to aerosolize fine particles for deposition in the lower respiratory tract [5,6]. In terms of fine powder production, in addition to traditional top-down milling processes, spray drying is a commonly used bottom-up technique that atomizes a feed liquid (such as a solution or a suspension) into fine droplets, followed by rapid drying by the hot air/nitrogen gas [7,8,9]. However, the spray-dried (SD) powder may be amorphous and/or hygroscopic, which can absorb atmospheric moisture [10,11,12,13]. Moisture absorption can lead to crystallization of amorphous substances, which is one of the factors causing physical instabilities [11,14]. Moreover, moisture absorption can deteriorate the aerosol performance by increasing inter-particulate forces such as capillary forces in the powder, leading to particle aggregation and even particle fusion that negatively impacts the particle dispersibility [15,16,17]. Therefore, moisture protection is essential for the stability and performance of the SD inhalable powder.

Particle surface modification is a strategy to protect the particle from moisture during manufacturing and storage, thereby improving the stability. The spray drying process may achieve such particle surface modification. In spray drying, droplet drying leads to the formation of dried particles with a specific radial distribution, which indicates that certain components in the formulation may become enriched on the surface or diffuse into the center of the particle [7,18]. Such radial distribution of the particle composition results from multiple factors, including the evaporation rate of water and the properties of the solute, such as solubility, surface activity, and molecular weight [7]. Briefly, at the air–liquid interface of droplets, solutes are concentrated at the droplet surface as water evaporates, creating a concentration gradient that drives solutes to diffuse into the center of the droplet. If the droplet surface ends up descending faster than solute diffusion, the particle becomes hollow [7]. Conversely, the particle becomes dense if solute diffusion is faster than the descent of the droplet surface [7]. Hence, by leveraging the formulation composition and the process parameter, spray drying can be used to modify the particle surface properties, which can prevent particle agglomeration due to moisture absorption and improve the stability of SD DPI formulations.

Hydrophobic amino acids have been incorporated into SD DPI formulations to provide protection against moisture deterioration [19,20,21,22,23,24,25,26,27]. Hydrophobic amino acids have been demonstrated to be enriched on the particle surface during particle formation of spray drying [20,21,23,28,29,30,31,32,33,34,35]. The underlying mechanism is hypothesized to be related to their low aqueous solubility [7,36]. Low solubility of the amino acids allows them to reach saturation faster than other components included in the formulation as water evaporates during the drying process, leading to phase separation at the air–liquid interface of the droplet where the saturated phase further limits the mobility of the solute and thus results in surface enrichment [7]. At the air–liquid interface, such hydrophobic amino acids can orient their hydrophobic regions toward the air, thereby increasing the hydrophobicity on the particle surface [25,37].

While surface enrichment of these hydrophobic amino acids can enhance the physical stability of the DPI formulations, it has also been reported to affect the formulation behavior including in vitro dissolution. Raula et al. observed that coating salbutamol sulfate particles with leucine via physical vapor deposition slowed down the dissolution, which was attributed to the lower water solubility of leucine than salbutamol sulfate [38]. It should be noted that this study applied an aerosol flow reactor rather than spray drying to produce the particles. In another study, disodium cromoglycate (DSCG) was spray-dried with another type of hydrophobic excipient, sodium stearate (NaSt); they demonstrated that NaSt was enriched on the particle surface, and such enrichment reduced the dissolution rate of DSCG when there was 50% and 90% NaSt (*w*/*w*) in the composite powders [20]. However, there are very limited studies to report the impact of such enrichment on in vitro cellular uptake, another critical factor in the safety and efficacy of drug products, for DPI formulations. Vartiainen et al. investigated the drug permeation across the Calu-3 cell monolayer of the leucine-coated dry powder containing beclomethasone dipropionate or salbutamol sulphate produced via an aerosol flow reactor [39]. They observed that as compared with the physical mixture where the permeation of beclomethasone dipropionate was not detectable, the composite powder showed 0.7% cellular permeation. However, they did not explain if the minor increase in cellular permeation was attributed to the excipient addition. In terms of cellular permeation of salbutamol sulfate, the composite powder showed no difference from the physical mixture.

So far, as compared with the impact of surface enrichment of hydrophobic amino acids on the physical stability in terms of the aerosol performance, more investigations are needed to understand the impact of such enrichment on in vitro dissolution and in vitro cell uptake for the SD DPI formulations. The objective of the present study was to evaluate the impact of such surface enrichment on both the storage stability under high-humidity conditions and in vitro dissolution and cellular uptake of SD DPI powders. Colistin, a hygroscopic amorphous antibiotic (water solubility 50 mg/mL [40]), was spray-dried with typical amino acids, either L-leucine or trileucine. Leucine and trileucine are similar in chemical structures as trileucine contains three residues of leucine, but they are different in certain properties. Leucine is of higher water solubility (22 mg/mL at 25 °C) than trileucine (6.8 mg/mL at neutral pH/25 °C) [31,41]. Leucine has been reported to crystallize in most of the spray drying studies [20,28,36], while trileucine remains amorphous [42]. The comparison of these two amino acids against moisture deterioration in aerosol performance was investigated in the present study. The stability of the SD colistin powders against moisture was tested at 75% relative humidity (RH) with respect to the aerosol performance. Various solid characterizations were performed to investigate the particle properties. In addition, the SD colistin powders with acceptable moisture protection were further evaluated for in vitro dissolution and in vitro cellular uptake.

## 2. Methods and Materials

### 2.1. Material

Colistin sulfate (colistin; purity >= 19000 U/mg) and polymyxin B (purity of 8214 IU/mg) were supplied by BetaPharma Co., Ltd. (Wujiang, China). Dexamethasone, trileucine, and L-leucine were supplied by Sigma-Aldrich (St. Louis, MO, USA). Acetonitrile (ACN), ammonium sulfate, ammonium hydroxide, methanol (MeOH), phosphoric acid (H_3_PO_4_), and sodium sulfate, were obtained from Fisher Scientific (Waltham, MA, USA). Dulbecco’s Phosphate Buffered Saline (PBS, 1X), Hanks’ Balanced Salt Solution (HBSS) with calcium and magnesium (1X), Penicillin-Streptomycin (PenStrep) with 10,000 units/mL penicillin and 10,000 µg/mL streptomycin, 2.5% Trypsin (10X), Fetal Bovine Serum (FBS, certified), Roswell Park Memorial Institute 1640 Medium (RPMI 1640 Medium), and Insulin-Transferrin-Selenium (ITS, 100X) were purchased from Gibco (Life Technologies Corporation, Eugene, OR, USA).

### 2.2. Production of Dry Powder Formulations by Spray Drying

Feed solutions (Table 1) with a total solid content of 20 mg/mL were dissolved in water. A BUCHI spray dryer (B-290, BUCHI Labortechnik AG, Flawil, Switzerland) was applied to produce the dry powder. Feed solutions were pumped at a rate of 2 mL/min. The parameters for spray drying were shown as follows: inlet air temperature, 120 °C; aspirator, 35 m^3^/h; atomizer, 742 L/h (60 mm); and outlet air temperature, 60 °C. The supplied trileucine was dissolved in water with pH adjusted to 11 by ammonium hydroxide [31] and spray-dried under the same parameters to increase the water solubility. The spray-dried trileucine was utilized to prepare the Col-Trileu formulations.

### 2.3. Storage Stability Test

The SD formulations (Table 1) were placed in 20 mL scintillation vials, left uncapped, and stored in a sealed stability container at 25 °C (room temperature) for one week. To maintain 75% RH, a saturated sodium chloride solution was prepared by mixing excess sodium chloride with water and placed at the bottom of the stability container.

### 2.4. Powder X-Ray Diffraction (PXRD)

A Rigaku Smartlab™ diffractometer (Rigaku Americas, The Woodlands, TX, USA) with a Cu-Kα radiation source and a D/tex ultra-detector was utilized to measure the powder X-ray diffraction pattern. The radiation source was operated at 40 kV voltage and 44 mA current. The settings were as follows: 5–40° 2θ at a step size of 0.02° with a scan rate of 4°/min [43].

### 2.5. Particle Size Distribution

An Aero S equipped Mastersizer 3000 (Malvern Panalytical, Malvern, UK) was used to measure the particle size distributions of the spray-dried samples. Each sample (approximately 50 mg) was dispersed using compressed air at 4 bars. The sizes below 10% (D_10_), 50% (D_50_), 90% (D_90_), and Span (calculated as [D_90_ − D_10_]/D_50_) were determined by the built-in software (version 3.62).

### 2.6. Scanning Electron Microscopy (SEM)

The particle morphology of the SD formulations was examined using a scanning electron microscope (NOVA nanoSEM, FEI Company, Hillsboro, OR, USA). A thin layer of the SD sample was placed on a stub with an adhesive carbon tape, followed by treatment of pressurized air to remove excessive particles. The particles were subsequently coated with platinum by sputtering at 40 mA for 2 min (208 HR, Cressington Sputter Coater, Watford, UK). The coated samples were imaged using the electron microscope at 5 kV acceleration voltage.

### 2.7. Dynamic Vapor Sorption

A dynamic vapor sorption (DVS) instrument (DVS Intrinsic, Surface Measurement Systems Ltd., London, UK) was utilized to characterize the isotherm moisture sorption/desorption behavior of the SD formulations. Approximately 8 mg of each formulation was used in the measurement. The SD powder was exposed to two cycles of an adsorption–desorption program: 0–90% RH with 10% steps at 25 °C. The percentage of the mass change as compared with the sample weight at 0% RH was recorded at each step, which indicated the adsorption or desorption of moisture by the sample. The equilibration for each step was controlled by dm/dt (%/min), which indicated the weight change per time. The dm/dt (%/min) was set as 0.002000 with the maximum duration time of 360 min. The data were presented as a plot of change in mass (%) vs. target RH (%).

### 2.8. X-Ray Photoelectron Spectroscopy

X-ray photoelectron spectroscopy (XPS) was performed via the K-Alpha X-ray Photoelectron Spectrometer System (Thermo Fisher, Waltham, MA, USA). The powders were pumped down for 2 h prior to scanning. The scan conditions were as follows: pass energy of 200 eV, 8 scans, dwell time of 10 ms, and step size of 1.0 eV. XPS is a technique that detects elements in the samples with a typical depth of 2–10 nm [44]. Thus, the surface composition can be investigated by XPS.

### 2.9. Assay of Colistin

High-performance liquid chromatography (HPLC) equipped with an Agilent Exclipse Plus C18 column was used to measure colistin for evaluating the in vitro aerosol performance and dissolution of the formulations [45]. Chromatographic separation was carried out at a flow rate of 1 mL/min, using an isocratic mobile phase comprising 30 mM Na_2_SO_4_, adjusted to pH 2.5 with H_3_PO_4_, and ACN in a 76:24 (*v*/*v*) ratio.

Colistin quantification in the cell-based experiments was performed using LC–MS/MS with an Agilent 6460 Triple Quadrupole system [45]. The chromatographic separation was conducted using a Kinetex C18 column (2.6 μm, 100 Å, 50 × 3 mm; Phenomenex, Torrance, CA, USA) with a gradient mobile phase consisting of ACN and water containing 0.1% *v*/*v* formic acid [45]. The gradient profile was as follows: 10% ACN (0–1 min), 70% ACN (5 min), 90% ACN (6–6.5 min), and 10% ACN (7.5–12 min), with a flow rate of 0.5 mL/min [45]. The internal standard for colistin detection was polymyxin B. Multiple reaction monitoring (MRM) was employed to analyze each drug with the following *m*/*z* transitions: colistin A (585.5–101.1), colistin B (578.5–101.1), polymyxin B1 (602.3–101.1), and polymyxin B2 (595.4–101.1). The operating conditions were set as follows: gas temperature at 350 °C, sheath gas temperature at 300 °C, nebulizer pressure at 35 psi, gas flow at 9 L/min, sheath gas flow at 9 L/min, nozzle voltage at 1000 V, and capillary voltage at 4000 V.

### 2.10. In Vitro Aerosolization Performance

A next-generation impactor (NGI, Copley, Nottingham, UK) was applied to evaluate in vitro aerosolization behavior of the SD formulations before and after exposure at 75% RH for a week. For each formulation, around 15 mg was weighed and transferred into a capsule (size-3 hydroxypropyl methylcellulose, Qualicaps, Whitsett, NC, USA). For each replicate (n = 3), two capsules (around 30 mg formulations) were aerosolized using a low-resistance RS01 DPI device (Plastiape S.p.A., Osnago, Italy). For actuation, vacuum pumps allowed 4 L of air passing through the instrument at a flow rate of 90 L/min, which generated a pressure drop of 4 kPa. The dispersed powders were collected with water from the capsule, device, NGI throat, and all the NGI stages.

### 2.11. In Vitro Dissolution

A diffusion Franz cell (V6B, PermeGear Inc., Hellertown, PA, USA) was employed to perform the in vitro dissolution test. A quantity of 20 mL of degassed PBS at pH 7.4 was loaded into the Franz cell as the dissolution medium and maintained at 37 °C [46,47,48] with stirring at 600 rpm (6-station Franz Cell stirrer, PermeGear Inc., Hellertown, PA, USA). The SD powder was dispersed onto a filter disk (Whatman^®^ Grade 2, pore size 5 μm, GE Healthcare, Parramatta, Australia) placed at stage 3 of the NGI. For each formulation, approximately 15 mg of the powder was weighed and loaded into a size 3 hydroxypropyl methylcellulose capsule. Two capsules (a total of 30 mg) were actuated for each replicate (n = 4). The filter disk with the dispersed powder was placed on the top of the Franz cell in contact with the dissolution medium. An aliquot of 150 µL sample was taken at 5 min, 10 min, 20 min, 30 min, 60 min, 90 min, and 120 min with an equal volume of water added back. Samples were subjected to HPLC analysis for quantification of colistin.

### 2.12. Cell Culture

The NCI-H441 (H441) cells were supplied by American Type Culture Collection (ATCC, Manassas, VA, USA). RPMI 1640 medium containing 10% (*v*/*v*) fetal bovine serum, penicillin (100 U/mL), and streptomycin (100 μg/mL) was the culture medium. The cells were maintained in a humidified incubator at 37 °C with 95% air and 5% CO_2_. Passaging was performed when the cells reached approximately 80% confluence, and detachment was achieved using trypsin. The “polarization” medium used in the culture of the H441 epithelium model was the culturing medium with 1% ITS (100X) and 1 µM dexamethasone [49].

### 2.13. In Vitro Cellular Transport

The H441 lung epithelium model was grown in 6.5 mm Transwell^®^ inserts with 0.4 µm pore polyester membranes (Corning Incorporated, Corning, NY, USA). H441 cells were seeded in the Transwell plate at a density of 2.5 × 10^5^ cells/cm^2^ in the culturing medium. After 24 h, the culturing medium was replaced by the polarization medium for both apical and basolateral chambers. On day 3, the medium in the apical chamber was removed and left empty. Only the medium in the basolateral chamber was replaced with the fresh polarization medium (600 µL) every other day. The cells were then cultured at the air–liquid interfaced (ALI) condition for 12 days before conducting the transport experiment.

**Figure 1:**
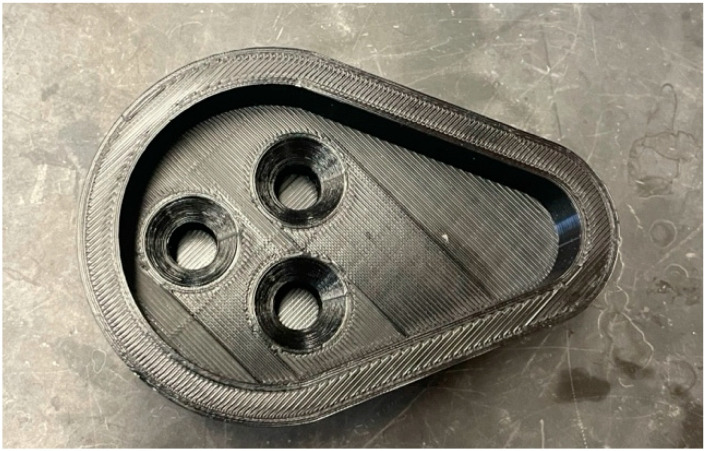
Modified NGI plate.

For the transport study, the H441 model was washed once with pre-warmed HBSS and allowed to equilibrate for 15 min in the pre-warmed HBSS in the incubator. The transepithelial electrical resistance (TEER) was assessed both prior to and following the transport study. Next, three Transwell inserts were transferred to a modified NGI plate, shown in Figure 1. The modified plate was placed at stage 3 in NGI to receive the dispersed powder. A quantity of 10 mg of each SD formulation was weighed and filled into a size 3 capsule. Two capsules (a total of 20 mg formulation) were actuated for one measurement. A 24-well polypropylene plate (Caplugs Evergreen, Buffalo, NY, USA) was used to conduct the transport study. Each well was filled with 600 µL of pre-warmed HBSS as the basolateral medium, and the Transwell inserts were placed to ensure the bottom of the membrane was in contact with the medium. Polypropylene plates were reported to mitigate the colistin adherence to the materials and reduce drug loss more than polystyrene plates [50]. At the end of the 4 h transport study, to collect any remaining powder on the cell surface, 100 µL of pre-warmed HBSS was used to rinse the apical chamber. The 600 µL basolateral HBSS was collected to determine the amount of the transported colistin. Samples were subjected to quantification of colistin by LC-MS/MS QQQ.

The intracellular drug content was evaluated based on the procedure outlined in prior research [45]. After the 4 h transport study, the Transwell membrane with the attached cells was rinsed twice with HBSS, removed, and placed into a 1.5 mL tube. To lyse the cells and extract the intracellular drug, 400 µL of RIPA buffer (Thermo Fisher, Waltham, MA, USA) was added, and the tube was incubated on ice for 10–15 min. This was followed by centrifugation at 15,000 g for 15 min at 4 °C. Subsequently, 100 µL of the RIPA supernatant was combined with 100 µL ACN and 100 µL MeOH and vortexed for 1 min to precipitate proteins, and the drug concentration was measured using LC–MS/MS QQQ.

The results were presented as the percentage of the colistin amount relative to the total drug recovered after the 4 h transport among three chambers, which were the apical chamber/remaining drug, basolateral chamber/transported drug, and cellular drug.

### 2.14. Statistical Analysis

Data were presented as mean values with standard deviations, based on at least three replicates. Microsoft Excel (version 16.61.1, 2022) was used for data analysis, while GraphPad Prism (version 10.0) was utilized for figure preparation and statistical analysis.

## 3. Results

### 3.1. Particle Size

Table 2 is a summary of the particle sizes of the SD formulations. All D_50_ values in these formulations were smaller than 3 µm. With the same solid content (20 mg/mL) during spray drying, the difference of the formulation compositions did not drastically change the D_50_; all D_50_ values were in the range of 2.0 to 2.5 µm. D_90_ values were below 5.5 µm, indicating that the majority of the particles had an aerodynamic diameter of less than 5 µm.

### 3.2. Particle Morphology

**Figure 2:**
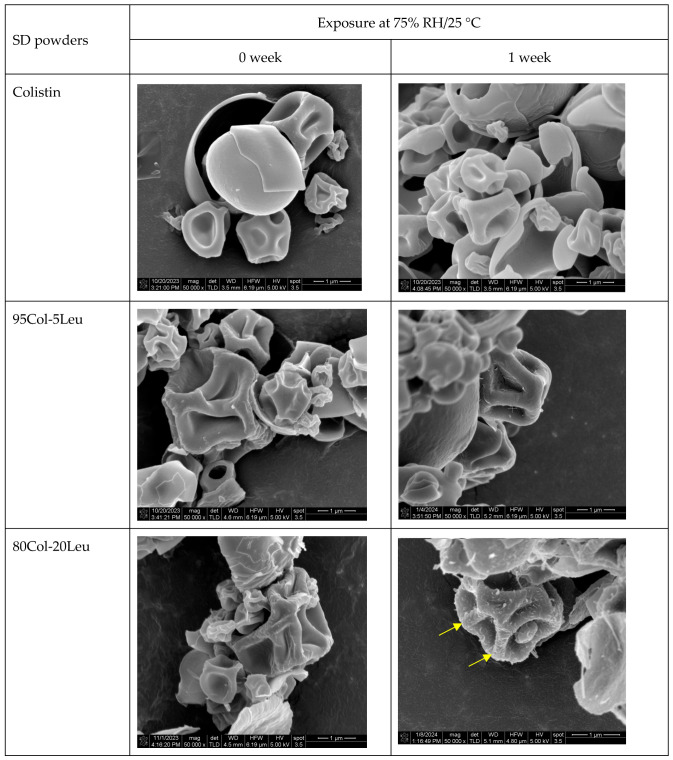

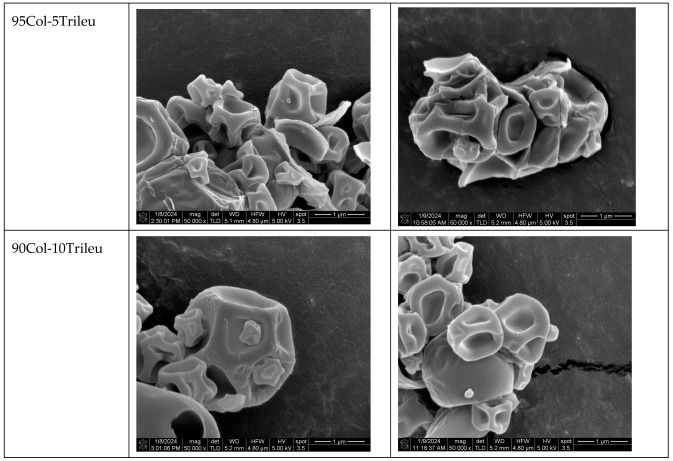
SEM images of the SD formulations.

Figure 2 shows the representative particle morphology of the SD formulations before and after exposure to 75% RH for a week. The addition of leucine or trileucine in the formulations showed a slight increase in surface corrugation as compared with SD colistin (0 week in Figure 2). After one-week exposure at 75% RH, the surface of Col-Leu powders showed morphology change indicated by the yellow arrows, whereas SEM images did not show any difference in the SD Col-Trileu powders or SD colistin powder. The morphology changes in the SD Col-Leu formulations were hypothesized to be leucine crystals, based on a previous study where they observed a similar morphology change on the SD particle surface containing leucine after moisture absorption [20].

### 3.3. Crystallinity

**Figure 3:**
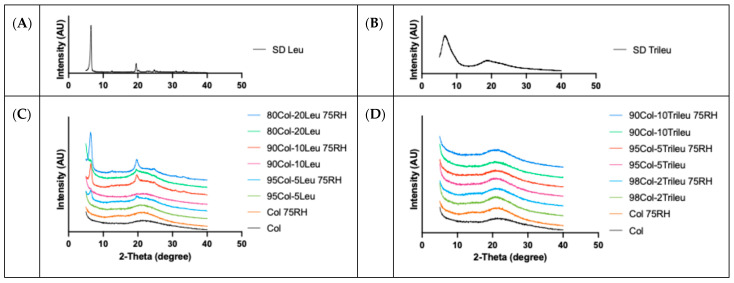
X-ray powder diffraction patterns of the SD formulations: (**A**) SD leucine, (**B**) SD trileucine, (**C**) SD colistin and SD Col-Leu formulations before and after one-week exposure at 75% RH, and (**D**) SD colistin and SD Col-Trileu formulations before and after one-week exposure at 75% RH.

Figure 3 is a summary of PXRD diagrams for the SD formulations, SD leucine, and SD trileucine. Leucine and trileucine showed crystallization tendencies after the SD process (Figure 3A,B). The SD colistin formulation showed no peaks, indicating that it was amorphous before and after exposure at 75% RH for a week. Regarding the SD Col-Leu formulations (Figure 3C), an increase in the amount of leucine resulted in peaks of higher intensity, with the peak locations aligning with those observed in the SD leucine (Figure 3A), implying that these peaks originated from leucine crystals. Such crystallization of leucine in spray drying has been reported elsewhere [20,28]. In addition, the intensity of such peaks increased after exposure to the high humidity in the same SD Col-Leu formulation, suggesting the presence of moisture-induced crystallization of leucine during the storage. The increase in the leucine crystallinity was consistent with the result by SEM (Figure 2). In the SD Col-Trileu formulations, no obvious peaks were observed, suggesting their being amorphous before and after the exposure at 75% RH.

### 3.4. DVS

**Figure 4:**
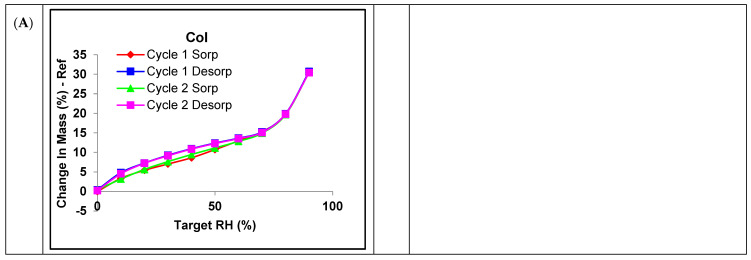

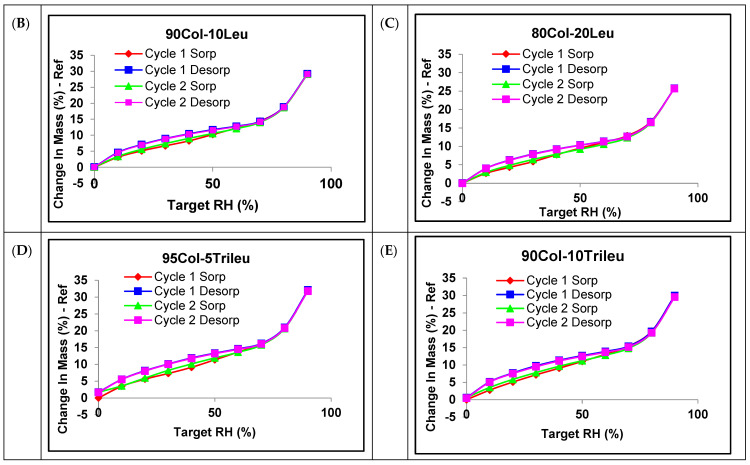
Characteristics of the dynamic vapor sorption over two Sorp-Desorp cycles for the SD formulations: (**A**) SD colistin, (**B**) 90Col-10Leu, (**C**) 80Col-20Leu, (**D**) 95Col-5Trileu, and (**E**) 90Col-10Trileu.

The SD colistin showed a mass increase of approximately 30% due to moisture adsorption when equilibrated at 90% RH (Figure 4A), suggesting its hygroscopicity [10,51]. In all formulations, there was a desorption hysteresis. Such slower desorption of absorbed water probably resulted from the water trapped inside the particle, which took longer to escape. In addition, there was no moisture-induced recrystallization. In the SD Col-Leu formulations, the moisture absorption equilibrated at 90% RH decreased by ~5% in the 80Col-20Leu as compared with the SD colistin (Figure 4C). The other Col-Leu formulations with a lower amount of leucine did not show significant differences in terms of moisture adsorption. In all the SD Col-Trileu formulations (Figure 4D,E), the moisture adsorption equilibrated at 90% RH was not changed significantly by the presence of trileucine compared with the SD colistin. In addition, upon one-week exposure to 75% RH, the SD colistin, 80Col-20Leu, and 90Col-10Trileu were analyzed by DVS again (Supplementary Figure S1), where the water sorption/desoption behaviors were not changed as compared with the 0-week samples.

### 3.5. XPS

Surface composition analyzed by XPS is summarized in Table 3. Since the sulfur element is uniquely present in colistin sulfate, the sulfur level can represent the level of colistin on the surface. Theoretical atomic percentages of sulfur were calculated based on the molecular formula of colistin B sulfate, C_104_H_206_N_32_O_46_S_5_. Theoretical values ([A] values in Table 3) assumed the surface concentration of colistin to be the same as the sample composition. All experimental values were normalized such that the experimental and theoretical values for the SD colistin were identical ([B] values in Table 3). In a comparison of normalized experimental value with the theoretical value in the sulfur atomic percentage, the experimental value was lower than the theoretical value for all SD Col-Leu and Col-Trileu, which suggested that the colistin was less present on the surface than the composition. In other words, leucine and trileucine were enriched on the particle surface during spray drying. An increase in leucine or trileucine led to better surface enrichment efficiency. In a comparison of leucine vs. trileucine, trileucine showed a better reduction in colistin coverage on the particle surface ([C] values in Table 3).

### 3.6. In Vitro Aerosol Performance

**Figure 5:**
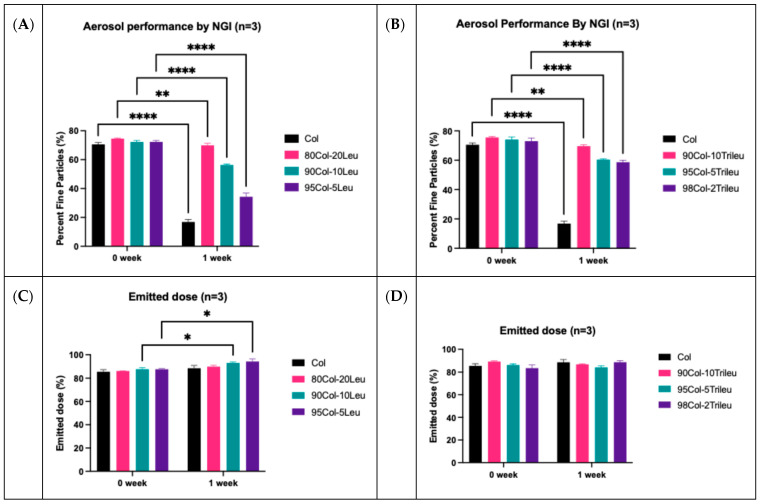
Summary of the fine particle fraction (FPF) and emitted dose as measured by NGI for the SD formulations before and after one-week exposure at 75% RH (mean ± SD, n = 3; * *p* < 0.05, ** *p* < 0.01, **** *p* < 0.0001, assessed by two-way ANOVA multiple comparisons using GraphPad Prism Version 10.0): (**A**) FPF of SD colistin and SD Col-Leu powders. (**B**) FPF of SD colistin and SD Col-Trileu powders. (**C**) Emitted dose of SD colistin and SD Col-Leu powders. (**D**) Emitted dose of SD colistin and SD Col-Trileu powders.

Figure 5 is a summary of the fine particle fraction (FPF) and emitted dose from the SD formulations before and after exposure at 75% RH for a week. Table 4 is a summary of FPF reduction (%) over the one-week period. FPF represents the fraction of particles with an aerodynamic diameter less than 5 µm. In all the SD formulations, FPF showed a statistically significant decrease (*p* < 0.01) after one-week exposure at 75% RH (Figure 5A,B). However, it should be noted that the FPF reduction (%; Table 4) for the 80Col-20Leu over the one-week exposure to 75% RH was approximately 6%. Despite the statistically significant difference (*p* < 0.01) in FPF due to the small standard deviation, the real difference was not substantial in 80Col-20Leu. A similar observation was also made for 90Col-10Trileu, where the FPF reduction (%; Table 4) was 7.87%. Therefore, 80Col-20Leu and 90Col-10Trileu were considered to provide acceptable moisture protection for SD colistin formulations and were further chosen to be evaluated for in vitro dissolution and cellular uptake. The emitted dose was the fraction of powders collected from stages 1 to 8 in NGI. In the Col-Leu powders (Figure 5C), the emitted dose showed statistically significant increases (*p* < 0.05) in both 90Col-10Leu and 95Col-5Leu powders over the one-week exposure. The emitted dose was not changed significantly in any of the Col-Trileu powders (Figure 5D).

### 3.7. In Vitro Dissolution

**Figure 6:**
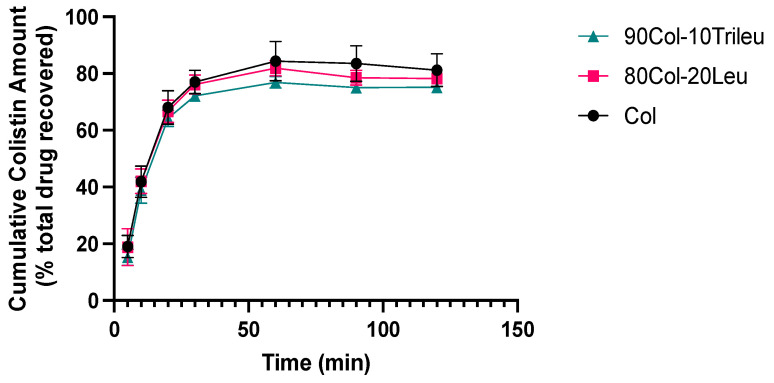
In vitro dissolution profile (mean ± SD, n = 4).

Based on the results of in vitro aerosol performance, 90Col-10Trileu and 80Col-20Leu were further evaluated regarding in vitro dissolution as compared with the SD colistin due to their acceptable moisture protection for the aerosol performance. In Figure 6, the dissolution profile did not exhibit any statistically significant difference among these formulations, suggesting that surface enrichment of leucine or trileucine did not affect in vitro dissolution of colistin. However, it should be noted that in vitro dissolution behavior may not fully represent in vivo dissolution profiles of inhalation formulations.

### 3.8. In Vitro Cell Transport

**Figure 7:**
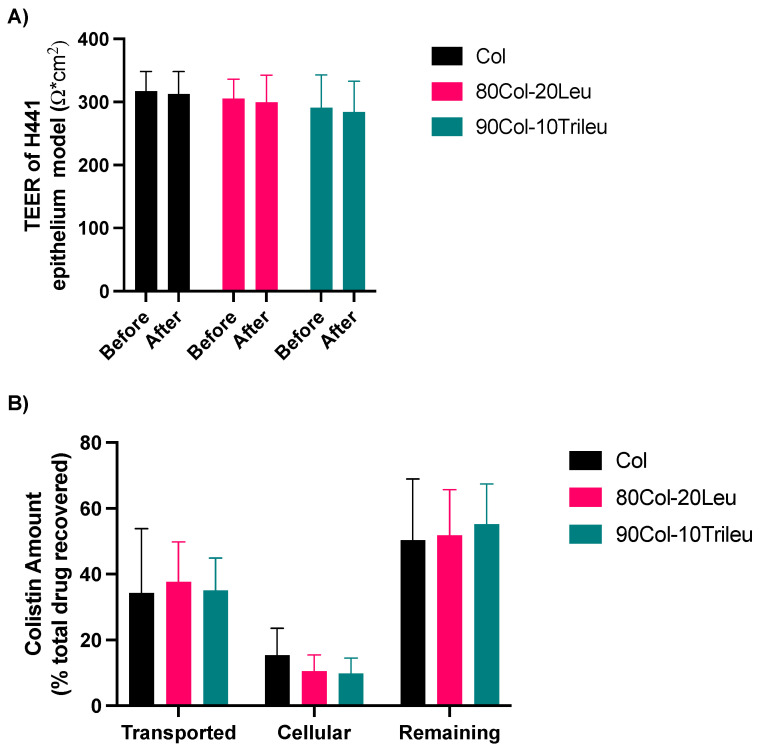
In vitro cell transport: (**A**) TEER values of the H441 epithelium model (mean ± SD, n = 18). (**B**) The amount of colistin relative to the total recovered drug among the three chambers (mean ± SD, n = 20).

Figure 7A shows TEER values of the H441 epithelium model before and after the 4 h transport. TEER values did not show statistically significant difference, indicating the integrity of the epithelium model throughout the transport experiment. Figure 7B shows the distribution of colistin relative to the total recovered colistin among the three compartments (remaining, cellular, and transported drug). The results showed that the surface enrichment of leucine or trileucine did not significantly change the in vitro cellular behaviors, including uptake or transport. In terms of the distribution, the cellular compartment showed the least accumulation of colistin. The transported colistin from these SD formulations was approximately 35% out of the total recovered colistin. The remaining colistin was roughly 50% out of the total recovered drug.

## 4. Discussion

The present study comprehensively evaluated the impact of surface enrichment by leucine or trileucine on the SD colistin formulations regarding aerosol performance, in vitro dissolution, and in vitro cellular uptake. Our results indicated that hygroscopic SD colistin was prone to moisture deterioration in terms of aerosol performance (Figure 5), and incorporation of leucine and trileucine provided moisture resistance for SD colistin. To investigate the moisture protection by leucine or trileucine, various solid characterizations were performed.

In terms of the SD Col-Leu powders, leucine demonstrated the ability to enrich on the surface and thus lowered the surface concentration of colistin (Table 3). The effect of such enrichment by leucine in spray drying has been reported elsewhere [20,21,28,29,30,32,33,35]. Since colistin is of higher water solubility than leucine, leucine could first reach the saturation and precipitate into the saturated phase of a low molecular diffusion rate, leading to the surface enrichment of leucine during spray drying. The enrichment of leucine in the SD colistin formulations was observed to increase particle surface corrugation (Figure 2), a phenomenon that has also been reported in previous studies [32,37].

Additionally, our results suggested that leucine was crystallized after spray drying (Figure 3), which was consistent with previous studies [20,28,37,52]. Since crystalline materials tend to absorb less water than amorphous ones [53], we further investigated the moisture uptake of these SD powders (Figure 4). Only 80Col-20Leu reduced the water uptake of SD colistin by approximately 5% when equilibrated at 90% RH. This indicated that the high leucine content could further reduce the water uptake. Moreover, upon one-week exposure at 75% RH, the increase in the crystal peak intensity of leucine (Figure 3) along with more leucine crystals observed under SEM (Figure 2) could be attributed to the water-induced leucine crystallization, where water absorption can increase the molecular mobility and rate of crystallization [54]. This also indicated that after spray drying, leucine was likely partially ordered instead of fully crystallized, which was consistent with a previous study [55]. Overall, our results suggested that the moisture protection of leucine in SD powders depended on the leucine concentration in the formulation. This protective effect can be attributed to (1) the particle surface enrichment of leucine and (2) a reduction in water uptake in SD formulations containing a high leucine content.

In the SD Col-Trileu powders, trileucine was found to increase the surface corrugation (Figure 2) and be enriched on the particle surface (Table 3), which was consistent with previous findings [31,56]. However, in comparison with leucine, trileucine, an oligopeptide being more conformationally flexible than leucine, was less observed to crystallize during spray drying [7,26,30]. Consistently, only the SD trileucine exhibited some degree of crystallinity but not in any of the Col-Trileu formulations (Figure 3). Additionally, colistin might delay and/or inhibit the crystallization of trileucine. The inhibiting effect of crystallization by colistin has been reported in a co-spray-dried colistin-ciprofloxacin formulation [43]. Furthermore, the presence of amorphous trileucine did not reduce the water uptake of SD colistin at 90% RH (Figure 4). Although trileucine was amorphous and did not reduce water uptake, the results of aerosol performance (Figure 5) indicated that trileucine outperformed leucine in terms of moisture protection. Ten percent trileucine was used as the highest ratio due to the low water solubility, but it was sufficient to provide moisture protection as compared with 80Col-20Leu (Figure 5B). Such superior performance could be attributed to the better surface enrichment efficiency by trileucine (Table 3). Trileucine is of lower water solubility than leucine [30], leading to a faster precipitation in spray drying based on Peclet number [7]. A recent study supported that when spray drying protein with trehalose, leucine, and trileucine, trileucine tended to form a layer near the particle surface, whereas leucine was distributed closer to the particle core, suggesting that trileucine was more quickly precipitated into the saturated phase than leucine during spray drying [57]. In addition, it should be noted that the solubility of trileucine is pH-dependent, and the lowest solubility is at neutral pH [31]. In the present study, the feeding solution was prepared at neutral pH. Overall, the superior performance of trileucine in restoring the aerosol performance against moisture can be attributed to the better surface enrichment efficiency.

Furthermore, the impact of surface enrichment by leucine and trileucine was investigated on dissolution as well as cellular uptake. For both tests, we utilized the NGI instrument to facilitate the powder deposition, enabling the evaluation of the dissolution and cellular uptake of these spray-dried powders after aerosolization. In vitro dissolution of these SD formulations did not exhibit significant differences among each other (Figure 6). The cutoff diameter of the particle size deposited on stage 3 of the NGI under a flow rate of 90 L/min was 2.30 µm [58]. Such a small particle size might have contributed to the similar dissolution pattern among different formulations, regardless of the particle surface composition. In addition, the surface coverage of these hydrophobic amino acids was not 100% (Table 3). Moreover, our results showed that surface enrichment by leucine or trileucine did not significantly alter the cellular transport or uptake in the H441 epithelium model (Figure 7), where H441 cells were representative of the lung alveolar epithelium [49,59,60]. Since dissolution was not affected by leucine or trileucine and dissolution would be the first step for the solid formulation to be absorbed by the cells [61], it was likely that the cellular uptake and transport of colistin were not affected by the enrichment either due to similar reasons.

## 5. Conclusions

This study demonstrated that co-spray drying colistin with leucine and trileucine provided a protective effect against moisture-induced deterioration in aerosolization, attributed to the enrichment of leucine and trileucine on the particle surface. Twenty percent leucine (*w*/*w*) and 10% trileucine (*w*/*w*) offered similar and acceptable moisture protection. Trileucine, a less water-soluble amino acid than leucine, outperformed leucine in terms of moisture protection due to a better surface enrichment efficiency. Importantly, our results indicated that surface enrichment of leucine or trileucine did not significantly alter the in vitro dissolution or cellular uptake of colistin in the H441 epithelium model.

## Figures and Tables

**Table 1:** Composition of the SD formulations.

Formulation	Total Solid Content	Colistin (%, *w*/*w*)	Leucine (%, *w*/*w*)	Trileucine (%, *w*/*w*)
Colistin	20 mg/mL	100	-	-
95Col-5Leu		95	5	-
90Col-10Leu		90	10	-
80Col-20Leu		80	20	-
98Col-2Trileu		98	-	2
95Col-5Trileu		95	-	5
90Col-10Trileu		90	-	10

**Table 2:** Particle size distributions of the SD formulations (mean ± SD, n = 3).

Formulation	D_10_ (µm)	D_50_ (µm)	D_90_ (µm)	Span
Colistin	1.13 ± 0.01	2.41 ± 0.07	5.34 ± 0.21	1.74 ± 0.04
95Col-5Leu	1.08 ± 0.02	2.22 ± 0.05	4.81 ± 0.22	1.68 ± 0.06
90Col-10Leu	1.05 ± 0.01	2.21 ± 0.02	4.85 ± 0.10	1.72 ± 0.04
80Col-20Leu	1.05 ± 0.01	2.10 ± 0.02	4.22 ± 0.03	1.51 ± 0.00
98Col-2Trileu	1.09 ± 0.03	2.34 ± 0.11	5.19 ± 0.45	1.75 ± 0.11
95Col-5Trileu	1.05 ± 0.01	2.25 ± 0.03	5.06 ± 0.14	1.78 ± 0.03
90Col-10Trileu	1.08 ± 0.02	2.28 ± 0.05	5.04 ± 0.19	1.73 ± 0.04

**Table 3:** Atomic percentage of sulfur on the surface of the SD powders determined by XPS (mean ± SD, n = 3).

SD Powders	Atomic Percentage of Sulfur (%)		Percentage of Colistin Reduction (C) = 100% − B/A × 100%
	Theoretical Value (A)	Normalized Experimental Value (B)	
Colistin	2.67	2.67 ± 0.04	0.00 ± 1.46
95Col-5Leu	2.54	2.43 ± 0.03	4.25 ± 1.17
90Col-10Leu	2.40	2.29 ± 0.08	4.39 ± 3.19
80Col-20Leu	2.13	2.02 ± 0.01	5.14 ± 0.49
98Col-2Trileu	2.62	2.38 ± 0.09	9.13 ± 3.26
95Col-5Trileu	2.53	2.07 ± 0.03	18.29 ± 1.31
90Col-10Trileu	2.40	1.75 ± 0.08	26.81 ± 3.37

**Table 4:** FPF reduction over the one-week exposure to 75% RH (mean ± SD, n = 3).

SD Powders	FPF Reduction (%) = (FPF_0_week_ − FPF_1_week_)/ FPF_0_week_ × 100%
Colistin	76.16 ± 2.24
80Col-20Leu	6.23 ± 2.36
90Col-10Leu	22.00 ± 0.43
95Col-5Leu	52.63 ± 2.94
90Col-10Trileu	7.87 ± 1.16
95Col-5Trileu	18.41 ± 1.99
98Col-2Trileu	19.56 ± 3.78

## Data Availability

The data of the present study are available upon request to the corresponding author.

## Supplementary Materials

The following supporting information can be downloaded at https://www.mdpi.com/article/10.3390/pharmaceutics17020199/s1: Figure S1: Comparison of the SD formulations before and after one-week exposure to 75% RH in characteristics of the dynamic vapor sorption for the SD formulations over two Sorp-Desorp cycles.
