# Supplementary figures and images for: Impact of Co-Spray Drying with Leucine or Trileucine on Aerosol Performance, In Vitro Dissolution, and Cellular Uptake of Colistin Powder Formulations for Inhalation
Source: Pharmaceutics. 2025 Feb 5;17(2):199. doi: 10.3390/pharmaceutics17020199 (PMC11858924; doi:10.3390/pharmaceutics17020199)

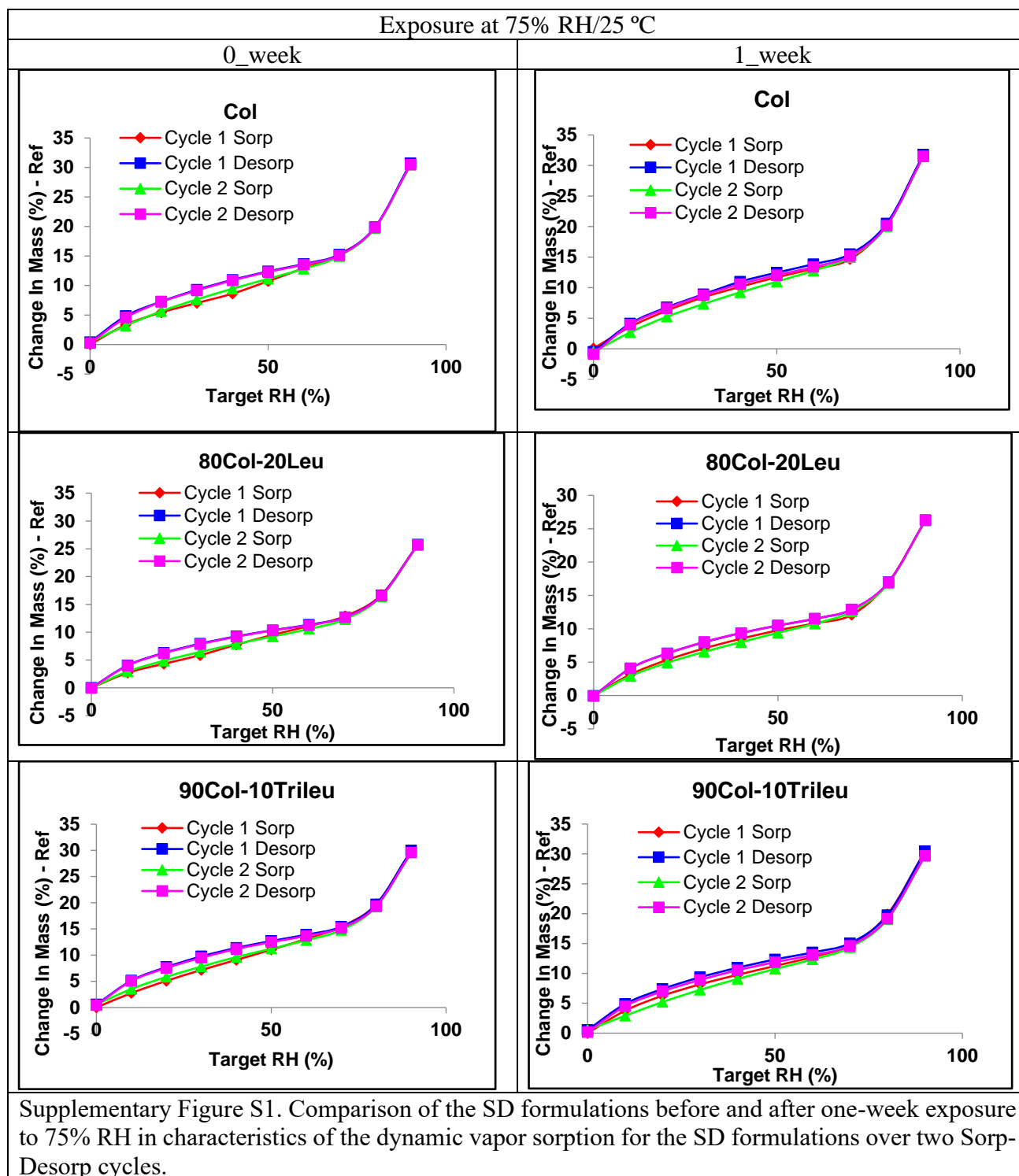

Supplement: Supplementary file 1 [file pharmaceutics-17-00199-s001.zip › pharmaceutics-3406325-supplementary.pdf]
